# Metabolic markers and microecological characteristics of tongue coating in patients with chronic gastritis

**DOI:** 10.1186/1472-6882-13-227

**Published:** 2013-09-17

**Authors:** Zhu-Mei Sun, Jie Zhao, Peng Qian, Yi-Qin Wang, Wei-Fei Zhang, Chun-Rong Guo, Xiao-Yan Pang, Shun-Chun Wang, Fu-Feng Li, Qi Li

**Affiliations:** 1 Shanghai University of Traditional Chinese Medicine, Cai Lun Road, Pu dong district, Shanghai 201203, China; 2 School of Life Sciences and Biotechnology, Shanghai Jiao Tong University, Life Sciences Building 800 Dong chuan Road, Shanghai 200240, China; 3 Department of Medical Oncology, Shuguang Hospital, Shanghai University of Traditional Chinese Medicine, Shanghai 201203, People's Republic of China

**Keywords:** Chronic gastritis, Tongue coating, 16S rRNA denatured gradient gel electrophoresis, Liquid chromatography and mass spectrometry

## Abstract

**Background:**

In Traditional Chinese Medicine (TCM), tongue diagnosis has been an important diagnostic method for the last 3000 years. Tongue diagnosis is a non-invasive, simple and valuable diagnostic tool. TCM treats the tongue coating on a very sensitive scale that reflects physiological and pathological changes in the organs, especially the spleen and stomach. Tongue coating can diagnose disease severity and determine the TCM syndrome (“Zheng” in Chinese). The biological bases of different tongue coating appearances are still poorly understood and lack systematic investigation at the molecular level.

**Methods:**

Tongue coating samples were collected from 70 chronic gastritis patients and 20 normal controls. 16S rRNA denatured gradient gel electrophoresis (16S rRNA–DGGE) and liquid chromatography and mass spectrometry (LC–MS) were designed to profile tongue coatings. The statistical techniques used were *principal component analysis* and partial least squares–discriminate analysis.

**Results:**

Ten potential metabolites or markers were found in chronic gastritis patients, including UDP-D-galactose, 3-ketolactose, and vitamin D2, based on LC–MS. Eight significantly different strips were observed in samples from chronic gastritis patients based on 16S rRNA–DGGE. Two strips, Strips 8 and 10, were selected for gene sequencing. Strip 10 sequencing showed a 100% similarity to *Rothia mucilaginosa*. Strip 8 sequencing showed a 96.2% similarity to *Moraxella catarrhalis*.

**Conclusions:**

Changes in glucose metabolism could possibly form the basis of tongue coating conformation in chronic gastritis patients. The study revealed important connections between metabolic components, microecological components and tongue coating in chronic gastritis patients. Compared with other diagnostic regimens, such as blood tests or tissue biopsies, tongue coating is more amenable to, and more convenient for, both patients and doctors.

## Background

Tongue diagnosis is a non-invasive, simple and valuable diagnostic tool, the use of which has been repeatedly affirmed by clinical practitioners of traditional Chinese medicine (TCM) for 3,000 years. TCM determines the appearance of the tongue to be an outer manifestation of the status of the human body. Tongue appearance considers the tongue coating and tongue body. The tongue coating refers to fur-like substances covering the surface of the human tongue, caused by processes of the spleen and stomach. TCM suggests that the tongue coating is a very sensitive index that reflects the physiological and pathological status of the organs, especially the spleen and the stomach. Xing Se Joan Mo said that “the tongue coating is formed by stomach (“stomach-Qi” in Chinese) and the five organs (“Wu-Zang” in Chinese) are all supplied by the stomach, so the tongue coating is the index of body status”. Clinical research has reported that the tongue body changes slowly in the development of chronic gastritis, but the tongue coating changes rapidly and obviously. The tongue coating can reflect the severity of the disease [1]. The study researched tongue coating metabolic markers and the composition of microorganisms in chronic gastritis patients to determine any relationship between tongue diagnosis (non-invasive detection) and metabolic processes or microorganisms.

In addition to the extensive practice of tongue diagnosis in TCM, modern technologies, especially tongue imaging analyses, have been introduced for tongue diagnosis [2-7]. Bai Jiang reported an important connection between the tongue coating microbiome and traditional tongue diagnosis, and illustrates the potential of the tongue coating microbiome as a novel holistic biomarker for characterizing patient subtypes by double-barcode 16S rRNA sequencing protocol [6].

Metabonomics is an emerging science that considers metabolites and variations of metabolic pathways in biological systems. Metabonomics shows high throughput, high sensitivity, and high accuracy. Metabonomics considers the human body as a whole system, which is consistent with the TCM concept (“Tian Ren He Yi” in Chinese), and also shows wide application prospects in TCM research [8,9]. Gao Jie reported specific metabolites in hepatocellular carcinoma patients using metabonomics; [10] and Zhao Hui reported that the levels of C-telopeptide, osteocalcin, and bone alkaline phosphatase increased in breast cancer patients using metabonomics [11].

Metabolic biomarker research has aroused considerable interest. However, metabolic samples are taken from blood, urine or tissue extracts. Compared with other diagnostic regimens, such as blood tests or tissue biopsies, tongue diagnosis is more amenable to, and more convenient for, patients as well as doctors.

We have previously researched the metabonomics of TCM greasy tongue coating [12]. The study investigated tongue coating metabolic markers of chronic gastritis patients to determine the relationship between tongue coating metabolites and chronic gastritis.

The tongue contains 10-fold more microorganisms than human cells in the intestine. These microorganisms play important functions in humans regardless of health, disease and drug metabolism. Many scientists consider that the human body’s physiological and pathological characteristics result from interactions between human gene and microbial gene populations. The 16S rRNA gene denatured gradient gel electrophoresis fingerprint technique (16S rRNA–DGGE) effectively shows complex microbial strips. The technique had been widely applied in various biological intestinal flora and environmental bacterium complex structure research. 16S rRNA–DGGE techniques can rapidly and accurately analyze the stability of samples, have good reproducibility and characterize the microbial composition of a DNA fingerprint. TCM tongue diagnosis shows potential applicable value for exploring the metabolic pathways and microecological changes of chronic gastritis.

## Methods

### Ethical statement

All samples were obtained as part of diagnostic criteria after patients gave written informed consent. The study was approved by the local ethics committee of Putuo Hospital, Shanghai, China (No.2012-32).

### Participant selection criteria

The participants of this study were mainly patients from Putuo Hospital, which is affiliated with Shanghai University of Traditional Chinese Medicine. All patients underwent a gastroscopy examination and were diagnosed with chronic gastritis. The people acting as normal controls were teachers or students from the Shanghai University of Traditional Chinese Medicine who completed regular physical examination reports and filled out health questionnaires [13]. Participants were divided into two groups: the chronic gastritis group (70 cases); and the normal control group (20 cases).

Diagnostic criteria for chronic gastritis were taken from The Digestive Disease Branch of Chinese Medical Association, Consensus from National Proseminar of Chronic Gastritis in 2000 [14]. Inclusion criteria were: (1) consistency with the diagnosis criteria for chronic gastritis; (2) confirmed by gastric endoscopy; and (3) aged between 20 and 75 years. Exclusion criteria were: (1) suffering from duodenal ulcer, gastric ulcer, gastric bleeding, or gastric or intestinal diseases; (2) suffering from disease of the liver, heart, kidney, lung, or brain, or any other organ, or having a psychiatric condition; (3) pregnant or lactating women; and (4) allergic constitution or multiple drug allergy sufferers. Samples from subjects who had a history of taking antibiotics within the last 1 month were excluded.

### Tongue coating samples

**Figure 1:**
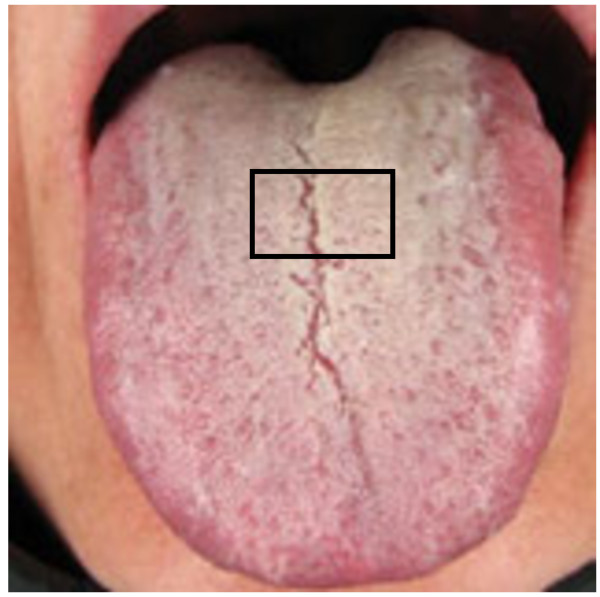
Sampling images of tongue coating from the centre of the tongue, an area regarded as tongue coating in the traditional tongue diagnosis.

All participants were required to gargle saline before sampling to rinse possible food contamination that might influence the tongue coating (Figure 1), which had got the patient’s informed consent. Small spoons were used to scrape the tongue coating and samples were placed into sanitized eppendorff tubes that had been filled with 2 ml of saline. Tongue coating samples were stored at room temperature for 1 to 2 h and then centrifuged for 10 min at 4°C at 1744 g. Epithelial cells of the tongue coating were counted and the epithelial cell concentration was adjusted to 10^6^ per ml saline. The supernatant was collected and stored at −80°C until analysis.

### Liquid chromatography–mass spectrometry (LC–MS) analysis

Tongue coating samples were prepared by centrifugation at 1744 g or 10 min. The supernatant was transferred to a 2 ml auto sampler vial equipped with a conical low-volume insert for analysis. A 150 μl aliquot of supernatant was added to 150 μl of acetonitrile and vibrated for 30 s. Samples were then centrifuged at 12000 g for 3 min before being transferred into a freeze drier. Freeze-dried samples were dissolved in 150 μl of 80% acetonitrile. In a typical experiment, a 20 μl aliquot of tongue coating was injected into a 2.1 mm × 100 mm HSS-T3 1.7 μm column using a Waters ZQ 2000 Series for LC–MS (Water, Massachusetts, USA). The column was maintained at 45°C and eluted with a linear gradient of 2% to 90% B at a flow rate of 600 μl/min (where A = 5 Mm AcNH4 + 0.1% FA and B = acetonitrile) for 0 to 10 min. After holding the solvent content to 100% methanol for 3 min, the column was returned to its starting condition. Column elution was separated so that approximately 250 μl/min eluent was introduced to electrospray ionization mass spectrometry.

Mass spectra were obtained on full-scan operation in positive ion mode. The capillary voltage was set at 3.6 kV, and the cone voltage was optimized at 30 V. The source temperature was set at 120°C, the desolation gas temperature was 300°C, and nebulization gas flow was 600 L/h. Data profiling of positive ions from m/z 70 to m/z 1000 was recorded at 1 s/scan during analysis. The tune mixture solution (Agilent Technologies, California,USA) was employed as the lock mass (m/z 118.09, 622.05 or 922.02) at a flow rate of 30 μL/min via a lock spray interface for accurate mass measurement.

### Date extraction

Typical total ion current chromatograms were unsuitable for pattern recognition because of overlapping peak profiles. However, significant visual differences were observed between patients with chronic gastritis and normal controls. The results of overall data analysis are presented in the flowchart. Prior to peak resolution, the LC–MS data, which comprised a two-way matrix (retention time × mass-to-charge ratio) for each sample, were exported and stored under the Analytical Instrumental Association. The output data were stored in a two-dimensional matrix. Retention time is included in one direction and mass-to-charge ratio (m/z) in another direction.

For LC–MS data, pattern recognition methods, principal *component analysis (PCA),* partial least squares–discriminate analysis (PLS–DA), and orthogonal partial least squares–discriminate analysis (OPLS–DA), were employed to identify the biochemical patterns in fur tongue and suggest variables that can be used as biomarkers for chronic gastritis.

### Polymerase chain reaction (PCR)–DGGE spectrum analysis of 16S rRNA V3 region

Bacterial genomic DNA extraction from 20 normal controls and 20 patients (cases with the thickest and greasiest tongue coating from 70 patients were selected) was performed. Tongue coating samples were washed with 0.1 M sodium phosphate buffer (pH 7.0) twice and then broken by the conventional bead-beater method [15]. Phenol chloroform was used to extract genomic DNA. The bacterial 16S rRNA gene V3 tongue variable regions of the amplification system have been discussed in the literature [16]. PCR is also available in the literature [17]. The 16S rRNA gene region V3 amplification products were concentrated and measured (PerkinElmer,Massachusetts,USA). We selected 200 V for complete gel electrophoresis (Bio-Rad,California,USA). Dyeing with SYBR Green was then performed. A gel imaging system (Tanon,Tokyo,Japan) and Image J were used to obtain the DGGE digitalized map for multivariate statistical analysis (PCA, PLS–DA) to determine any significant differences between the strips in each group.

Target strips for DGGE were cut using an aseptic operation blade and fragments were put in sterile double-distilled water at 4°C overnight. 16S rRNA V3 PCR gene amplification was then performed. The PCR reaction system and procedure were the same as previously described (above). After PCR products were collected using a gel extraction kit (Bio-Tek, Montpelier,USA) and purified, they were connected with pGEM-T Easy Vector (Promega, Wisconsin,USA) and transformed to accept sensitive cells using chemical technology (TransGen Biotech,Beijing,China). A white colony was selected and plasmid DNA was rapidly extracted using the 0.5% Triton-X100 cracking method for PCR amplification. PCR reaction conditions and reaction system were the same as previously described (above). Amplified PCR products were analyzed by DGGE and cloned similarly to those in the original tapping DGGE strip location.

## Results

### Clinical data

The study included 70 patients at Putuo Hospital between 2009 and 2010. The hospital is affiliated with Shanghai University of Traditional Chinese Medicine. Informed consent was obtained from all patients. Of the 70 cases (36 male, 24 female), 20 cases had atrophic gastritis and 50 cases had superficial gastritis. The mean age was 43.95 ± 13.36 years. The 20 cases that made up the normal controls included teachers and graduate students (9 male, 11 female). The mean age was 44.6 ± 14.78 years. No significant differences in gender and age were observed between the two groups (P > 0.05).

### Chromatographic analysis and comparison between the chronic gastritis group and normal controls

**Figure 2:**
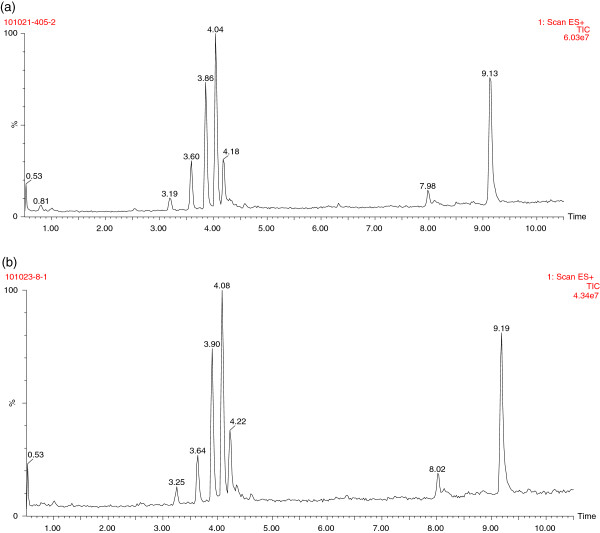
**Typical UPLC/MS metabolic fingerprinting total ion chromatogram of human tongue coating samples. (a)** the normal controls **(b)** the chronic gastritis group.

Typical base peak ion chromatograms from chronic gastritis patients and normal controls were compared. Differences in peak heights were observed between the two groups (Figure 2), which indicated that content and composition were different. The samples were then analyzed using multivariate statistic tools for further research.

### PCA and PLS–DA analysis of metabolic profiles of tongue coating samples from the chronic gastritis group and normal controls

**Figure 3:**
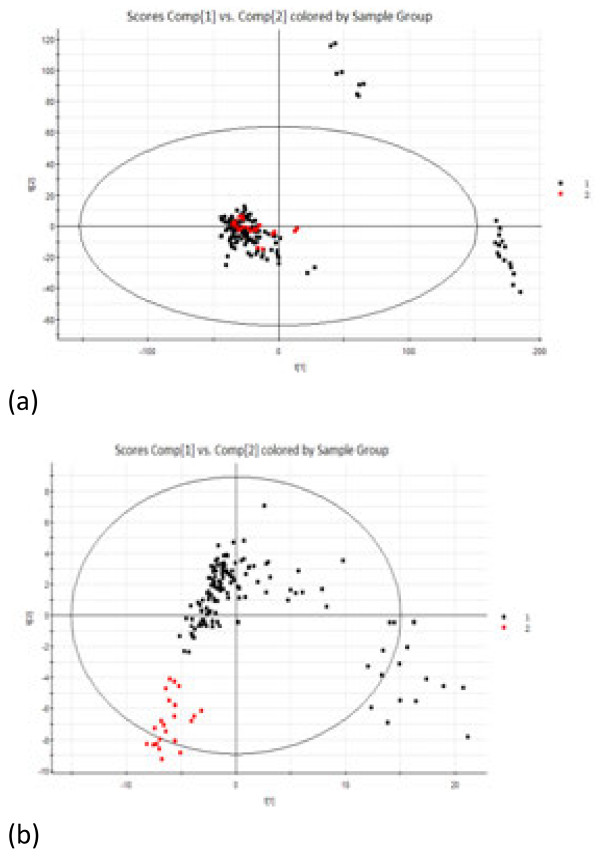
**PCA and PLS-DA analysis of the metabolic profiles of tongue coating samples from the chronic gastritis group and the normal group. (a)** PCA analysis **(b)** PLS-DA analysis, Red represents the normal group, and black represents the chronic gastritis group.

PCA appeared partially overlapped and mainly in the left quadrant. Further research by PLS–DA showed that the sample points were completely separated, which indicated that the two groups’ metabolic pathways were different (Figure 3). To improve the accuracy of the PLS discriminated model, OPLS–DA was used to analysis the results by removing some redundant information, such as environmental factors, gender, and diet.

### OPLS–DA comparison between the chronic gastritis group and normal controls

**Figure 4:**
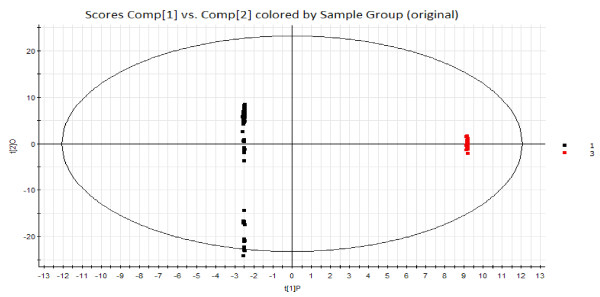
**Scoring chart by OPLS–DA analysis for the greasy coating group and the normal group.** Black denotes the chronic gastritis group, and red represents the normal group.

Tongue metabolic fingerprint differences between the chronic gastritis group and normal controls were compared using OPLS–DA. There were many different quadrants between the two groups (Figure 4). A large number of mass-to-charge ratios of metabolites were found based on the corresponding load diagram. We evaluated the potential biomarkers by variable importance projection (VIP). A total of 50 different metabolites were found between the two groups after the VIP value (VIP value > 2) was computed. Ten metabolites with the largest VIP values were identified and evaluated using a database and past biochemical studies of tongue coating cells (Table 1).

**Table 1:** Compounds and relative contents of the components of potential markers in the chronic gastritis group and the normal group

**t**_**R**_**/min**	**m/z**	**VIP**	**Compounds**	**Relative normal group**
3.59	566.0	2.38	UDP-D-galactose	↑
4.06	396.8	3.13	Vitamin D2	↓
4.07	340.1	3.25	3-Ketolactose	↑
4.08	379.2	3.39	Metarhodopsin	↑
8.14	334.2	3.59	Prostaglandin A2	↓
8.18	318	2.22	Leukotriene A4	↑
8.56	302.2	2.85	17alpha-methyltestosterone	↓
8.86	177.9	3.03	Pyrophosphate	↑
9.17	127	2.64	Piperideine-2-carboxylate	↓
9.25	310.0	3.00	Ribulose-1,5-bisphosphate	↓

### Comparison of 16S rRNA gene region V3 PCR–DGGE diagrams between the chronic gastritis group and normal controls

**Figure 5:**
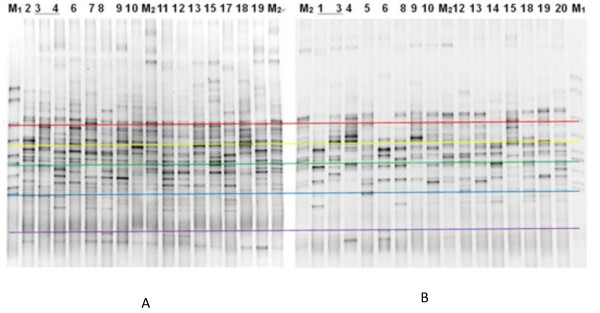
**PCR–DGGE atlas of 16S rRNA gene V3 region between the chronic gastritis group and the normal controls. (A)** Normal group, M1-marker, M2- control,2,34,6,7,8,9,10,11,12,13,15,17,18,19- normal groupsample. **(B)** Chronic gastritis group, M1-marker, M2-control,2,34,6,7,8,9,10,11,12,13,15,17,18,19- sample.

PCR products of the bacterial 16S rRNA gene V3 region from two groups of tongue coating samples were processed using DGGE. The atlas is shown in Figure 5. Examination of the DGGE atlas revealed numerous bacterial DGGE atlas strips and some main strips in the two groups.

**Figure 6:**
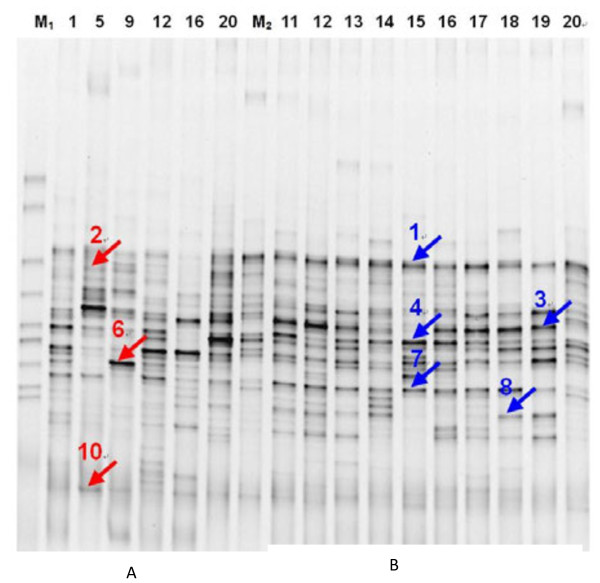
**Comparison of PCR–DGGE diagrams of 16S rRNA gene V3 region between the normal controls and the chronic gastritis group. (A)** Normal group, M1-marker, M2- control, normal group samples --1, 5, 9,12,16,20. **(B)** Chronic gastritis group, M1-marker, M2-control, Chronic gastritis samples --11,12,13,14,15,16,,1718,19,20.

**Figure 7:**
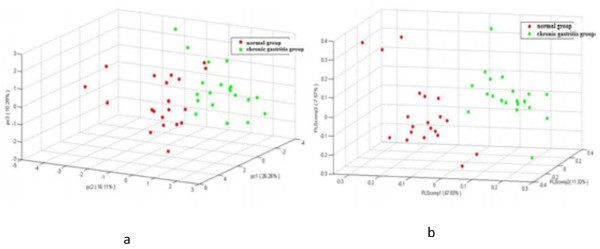
**PCA and PLS-DA of the 16S rRNA gene V3 region of the normal group and the chronic gastritis group. (a)** PCA results, red dots-normal samples, green dot- chronic gastritis patients **(b)** PLS-DA results, red dots-normal samples, green dot- chronic gastritis patients.

The DGGE atlases of the two groups were different (Figure 6). Bacteria of the types the strips represented may indicate an association with the occurrence and development of chronic gastritis—a possible association that requires further study. Eight strips from the two groups were markedly different (Figure 6). Strips 1, 3, 4, 7, and 8 were abundant in gastritis patient samples; whereas strips 2, 6, and 10 were abundant in normal samples. The bacteria that these strips represent can be associated with chronic gastritis. PCA of the data from the two groups revealed that the samples were generally located in two different regions (Figure 7(a)). When data from the two groups were set as the model, data was able to be separated (Figure 7(b)).

### DGGE strip sequencing results

Strips 8 and 10, the most markedly different strips among the samples, were selected for sequencing. Strip 8 sequencing indicated that its nearest neighbor was *Moraxella catarrhalis*; showing a 96.2% similarity. Strip 10 sequencing showed a 100% similarity with *Rothia mucilaginosa*.

## Discussion

Throughout the long history of traditional clinical practice in China and other Eastern countries, TCM practitioners have typically classified patients with the same disease into subgroups of different syndromes from a holistic perspective based on a patient’s overall status [6]. The appearance of the tongue coating is one of the major factors. The stage of a disease can be determined and treated by observing the tongue coating. In recent years, much tongue coating research had been conducted. In addition to the extensive practice of tongue diagnosis in TCM, modern technologies have been introduced for tongue diagnosis [18-25]. Tongue diagnosis in TCM shows potential applicable value for exploring metabolic pathways and microecological changes of chronic gastritis.

Chronic gastritis can cause changes to both the pathophysiology of a patient and their metabolites. By analyzing relationships between metabolites and diseases it is possible to identify biological markers. Kettunen et al. found 117 metabolic markers, including lipoprotein subclasses, amino acids, lipids and maximum genome-wide association, using the nuclear magnetic resonance high-throughput method [26]. They found 31 gene regions related to metabolite concentrations in the blood. Kleberg demonstrated that increased expression of PEAK1 could catalyze the proliferation of pancreatic tumor cells; identifying PEAK1 as a biomarker and a small-molecule therapeutics target [27].

Metabonomic studies of chronic gastritis are rarely reported. The determination of metabolic biomarkers from biological samples (blood, urine, tissue extract) can help distinguish chronic gastritis high-risk groups. The results of the present study aid in simplifying the clinical diagnosis process by providing information on early detection, early diagnosis and early treatment of the disease. LC–MS shows many advantages compared with other metabolomics methods [28]. For example, LC–MS has high resolution and sensitivity, special target metabolites can be detected within a short period, and it is possible to simultaneously analyze numerous compounds in a complex matrix.

Tongue coating metabonomics showed that potential markers were related to sugar metabolism, such as 3-keto lactose. UDP-D-galactose is a metabolic product of galactose and is involved in the synthesis of oligosaccharides. 2-deoxy-D-ribose is the basic raw material of RNA and DNA genetic material in the life system and is used as an ingredient in some vitamins and coenzymes.

Spleen governing transportation (“Pi Zhu Zhuan Yun” in Chinese) means that the spleen processes substances for transport to nourish the limbs and bones. Phlegm, blood stasis, indigestion, and other pathological factors often cause spleen and stomach disorders. The transpiration of stomach Qi changes the tongue coating, which indicates its possible relationship with abnormal energy circulation. These materials are mostly the products of energy metabolism, similar to the results of cell chemical structures [29]. A change in glucose metabolism is one of the mechanisms of tongue coating formation in patients with chronic gastritis, which suggests that the incidence of chronic gastritis is related is some way to energy metabolism changes.

Human ecology is an emerging branch of life science that mainly studies interactions and connections between normal flora and the host. The four major microecological areas in the human body are the oral cavity, gastrointestinal tract, skin, and vaginal tract. The human body has approximately 10^10^ bacteria; with 78% existed in the gastrointestinal tract. These bacteria interact with the body to achieve an inner balance. Once the balance is broken, bacterial pathogenesis occurs. One example is diarrhea, which is induced by intestinal dysbacteriosis. This pathogenesis is consistent with that in Chinese medicine.

Microecological study of the tongue coating has been frequently reported in recent years and has determined a close association between tongue coating and microorganisms. A study examining tongue coating found that spleen–stomach damp heat syndrome leads to a reduction in Gram-positive cocci and an increase in Gram-negative bacilli [30]. Yumin Sun et al. reported that the dominant bacteria in white coating and yellow coating were hemolytic streptococcus type A and catarrhal cuscus, whereas the dominant bacteria in white sticky coating and yellow sticky coating were hemolytic streptococcus type A and yellow pharyngeal bacteria [31]. Yue fei Jiang et al. reported that the diversity of flora on the tongue coating in patients with diarrhea-type irritable bowel syndrome and spleen–stomach damp heat syndrome was higher than that of the normal control group and the spleen-deficiency group [32]. Jing Wang et al. observed a microecological imbalance in the tongue coating of patients with acute pancreatitis, and changes in tongue coating were related to the severity of illness [33]. The amount of bacteria of the thick coating is more than that of the thin coating, which mainly consist of G-anaerobes. Anaerobic bacteria increase and aerobic bacteria decrease on the thick coating.

The present study included 20 cases of chronic gastritis and 20 normal controls to analyze the 16S rRNA gene V3 region by PCR–DGGE. DGGE mapping and multivariate statistical analysis indicated a difference in bacterial composition between the two groups of samples. The most different strips from these two groups were also identified by statistical analysis. The most pronounced strips (Strips 8 and 10) were then recycled and sequenced, and their nearest neighbors were determined as *M. catarrhalis* (*M. mora* bacteria) and *R. mucilaginosa*.

*R. mucilaginosa* is part of the normal flora existing in human nasopharyngeal [34], oropharynx, and upper respiratory tracts that can be isolated from the nasopharyngeal cavity and bronchial secretions. However, information on *R. mucilaginosa* is limited. The species was first reported in 1900 by Migula and was called *Micrococcus mucilaginosus* at the time. In 1907, the species was renamed as *Streptococcus salirarius* by Andrews and Gordon. *R. mucilaginosa* is a type of facultative anaerobic and Gram-positive coccus that comes in pairs or sets on smears, appears white in blood culture medium, and is non-hemolytic. *R. mucilaginosa* adheres to the surface of the culture medium and exists in the human oral cavity. This type of bacteria is considered an opportunistic pathogen. The bacterial catalyses is weakly positive or negative, and all enzymes and coagulates are negative. *R. mucilaginosa* is active in respiration and fermentation and can decompose galactose, fructose, glycerol, malt sugar, mannose, and sucrose, among others.

The results from the present study showed that brightness gradually decreased from the normal group to the chronic gastritis group. Preliminary cell chemistry experiments indicate that chronic gastritis occurs because of a broken balance in energy metabolism in tongue epithelial cells, activating the pentose shunt and enhancing oxidation. Consequently, unusual hyperplasia and differentiation of cell metabolism occur.

The tongue coating is composed of epithelial cells, bacteria, and food residues [26]. When epithelial cells are metabolically active, the amount of oxygen in the environment after metabolism can cause an accumulation of anaerobic bacteria. Facultative anaerobic bacteria perform aerobic and anaerobic fermentation simultaneously. These bacteria can grow in either aerobic or anaerobic environments but thrive better in an aerobic environment. Most pathogenic bacteria have such a nature. The facultative anaerobic bacteria detected in this experiment can decompose sugars, such as glucose, fructose, and galactose, which suggests that the formation of chronic gastritis is associated with sugar metabolism. Comparisons between microbial compositions from the chronic gastritis group and the normal group suggest that patients with chronic gastritis showed a relationship with inner energy metabolism and intestinal microflora changes. In addition, the brightness of the bacterial strip exhibiting 96.2% similarity with *M. catarrhalis* in the chronic gastritis group was higher than that in the normal control—which, to the best of the authors’ knowledge, is the first time such an observation has been reported. This kind of bacteria, which was closely related to the formation of chronic gastritis, deserves further investigation.

## Conclusions

We observed the metabolic components and microecological indexes of tongue coating in patients with chronic gastritis. The results of this study suggest that changes in metabolic patterns and microecological indexes were associated with chronic gastritis, and could be identified using the metabonomic method based on LC-MS analysis and microbiological techniques based on DGGE and multivariate statistics. PLS–DA and OPLS–DA score plots suggested distinct differences between the normal control group and the chronic gastritis groups. Nine specific variables and two bacterial strips were used in data analysis. The tongue coating indicates a close relationship between body energy metabolism and intestinal microflora changes, thus providing a theoretical basis for non-invasive diagnosis.

## Competing interests

The authors declare that they have no competing financial interests.

## Authors’ contributions

FFL and LQ conceived the idea and designed the research. XYP, YQW and CRG acquired and processed the data. J Z, SCW, WFZ and PQ performed the research and analyzed the results. ZMS, FFL wrote the paper. All authors read and approved the final manuscript.

## Pre-publication history

The pre-publication history for this paper can be accessed here:

http://www.biomedcentral.com/1472-6882/13/227/prepub
